# Association of Internet Use Frequency and Purpose with Subjective Well-Being in Japanese Older Adults: A Cross-Sectional Exploratory Study from the Chofu-Digital-Choju Project

**DOI:** 10.3390/ejihpe15100208

**Published:** 2025-10-12

**Authors:** Tsubasa Nakada, Kayo Kurotani, Satoshi Seino, Takako Kozawa, Shinichi Murota, Miki Eto, Junko Shimasawa, Yumiko Shimizu, Shinobu Tsurugano, Fuminori Katsukawa, Kazunori Sakamoto, Hironori Washizaki, Yo Ishigaki, Maki Sakamoto, Keiki Takadama, Keiji Yanai, Osamu Matsuo, Chiyoko Kameue, Hitomi Suzuki, Kazunori Ohkawara

**Affiliations:** 1 Graduate School of Informatics and Engineering, The University of Electro-Communications, Tokyo 182-8585, Japan; tsubasanakada@uec.ac.jp (T.N.); maki.sakamoto@uec.ac.jp (M.S.); yanai@cs.uec.ac.jp (K.Y.); ma004017@edu.cc.uec.ac.jp (O.M.); kameue.chiyoko@uec.ac.jp (C.K.); 2 Graduate School of Life Sciences, Showa Women’s University, Tokyo 154-8533, Japan; k-kurotani@swu.ac.jp; 3 Institute of Well-Being, Yamagata University, Yamagata 990-9585, Japan; seino.s@med.id.yamagata-u.ac.jp; 4 Faculty of Human Health, Komazawa Women’s University, Tokyo 206-8511, Japan; t-kozawa@komajo.ac.jp; 5 Faculty of Humanities and Social Sciences, Tokyo Metropolitan University, Tokyo 192-0397, Japan; shin1@tmu.ac.jp; 6 Faculty of Human Sciences, Osaka University of Economics, Osaka 533-8533, Japan; eto@osaka-ue.ac.jp; 7 School of Nursing, The Jikei University, Tokyo 182-8570, Japan; jshimasawa@jikei.ac.jp (J.S.); yumiko_shimizu@jikei.ac.jp (Y.S.); 8 Center for Health Sciences and Counseling, Kyushu University, Fukuoka 819-0395, Japan; tsurugano@chc.kyushu-u.ac.jp; 9 Sports Medicine Research Center, Keio University, Yokohama 223-8521, Japan; fuminori@keio.jp; 10 Green Computing Systems Research Organization, Waseda University, Tokyo 169-8050, Japan; exkazuu@gmail.com; 11 Faculty of Science and Engineering, School of Fundamental Science and Engineering, Waseda University, Tokyo 169-8050, Japan; washizaki@waseda.jp; 12 Research Center for Realizing Sustainable Societies, The University of Electro-Communications, Tokyo 182-8585, Japan; ishigaki@uec.ac.jp; 13 Information Technology Center, The University of Tokyo, Chiba 277-0882, Japan; takadama@g.ecc.u-tokyo.ac.jp; 14 Office for Research Management, The University of Electro-Communications, Tokyo 182-8585, Japan; suzuki.hitomi@uec.ac.jp

**Keywords:** internet use, WHO-5, subjective well-being, aging, digital divide, digital capital

## Abstract

The association between patterns of internet use for older adults’ well-being is unclear. We examined the association between the frequency and purpose of internet use and subjective well-being in older Japanese adults. We analyzed cross-sectional data from 2343 community-dwelling older adults (aged 65–84 years). Subjective well-being was measured using the World Health Organization Well-Being Index as a continuous score, and internet use was categorized by frequency and purpose. Hierarchical linear regression analysis was controlled for sociodemographic and health-related covariates. After full adjustment, only daily (B = 1.04, 95% CI [0.53, 1.56]) and dual-purpose use (i.e., for both practical and social communication purposes; B = 0.80, 95% CI [0.28, 1.31]) were independently associated with higher well-being. The analysis of the combined patterns further suggested that daily use was the primary factor. For older adults, regularity of internet use was more strongly associated with well-being than diversity of purpose. Daily integration appears to be a key factor for realizing benefits, suggesting that sustained practice is the foundational step in building the digital capital necessary for a flourishing later life. Longitudinal studies are needed to confirm these findings and untangle the causal relationship between sustained internet use and improved well-being among older adults.

## 1. Introduction

In an era of global population aging, maintaining and promoting the well-being of older adults is important for public health. Well-being is defined as a state in which individuals realize their abilities, cope with the normal stresses of life, function productively, contribute to their community, and find contentment (World Health Organization, 2013). Poor well-being can increase mortality risk among older adults (Chida & Steptoe, 2008; Rodda et al., 2011). Internet use has emerged as a key strategy for promoting the well-being of older adults. A growing body of evidence, including recent meta-analyses, indicates that online engagement is associated with positive outcomes, such as fewer depressive symptoms and higher life satisfaction (Liu et al., 2025; Luo et al., 2024). Thus, digital inclusion is a crucial component of healthy aging initiatives.

However, the benefits of internet use are not universally received. This disparity in outcomes, where some individuals benefit while others do not, is precisely what the evolving concept of the digital divide seeks to explain. This theory is divided into three levels: inequalities in physical access, digital skills, and the tangible real-world benefits that users derive from online activities (Ragnedda et al., 2020; Scheerder et al., 2017). To explain this gap in the results on well-being through internet use, a theoretical framework for “digital capital” may be indispensable. Digital capital is conceptualized as the sum of “internalized abilities and aptitudes (digital competencies)” and “externalized resources (digital technology),” which can be accumulated and converted into tangible life benefits (Ragnedda, 2018). From this perspective, achieving well-being is not merely about having access to the internet but also about effectively building and utilizing the digital capital needed to derive tangible benefits from its use.

The mechanism through which digital capital is thought to enhance well-being among older adults is by strengthening social connectedness and mitigating loneliness. Umbrella reviews have established that interventions utilizing digital technologies can effectively reduce social isolation and loneliness (Balki et al., 2022). Specifically, these technologies can facilitate communication with family and friends (Gunnes et al., 2024). Such enhanced social connections, in turn, are strong predictors of well-being (Heo et al., 2015; Szabo et al., 2019).

Digital capital acts as a bridge, allowing individuals to convert their digital activities into tangible real-world outcomes, including enhanced well-being. However, its practical formation remains unclear. Studies have investigated the frequency and purpose of online activities as separate entities (Lam et al., 2020; Vidiasratri & Bath, 2022). Nevertheless, although frequency and purpose are recognized as important, their relative contributions to well-being and the mechanics of digital capital formation remain poorly understood. Specifically, it is unclear whether the benefits of internet engagement for older adults stem more from regularity of use or diversity of purpose. This represents a critical gap in the literature, as clarifying this point is essential for designing effective digital inclusion initiatives. Understanding which regularity or diversity serves as the primary factor for well-being can help shape policies that foster the most beneficial patterns of internet use.

Accordingly, this exploratory study aimed to examine the independent and combined associations of internet use frequency and purpose diversity with subjective well-being among older adults in Japan. This study sought to provide a nuanced understanding of the digital behaviors that most effectively build the capital necessary for a flourishing later life. It should be noted that the cross-sectional design of this study precludes any causal inference.

## 2. Materials and Methods

### 2.1. Study Population

A cross-sectional analysis was conducted using baseline data from the “Chofu–Digital–Choju” (CDC; “Choju” means “longevity” in Japanese) project, a community-based intervention aimed at promoting health among community-dwelling older adults in Chofu City, Tokyo. The detailed methodology of this project has been described elsewhere (Nakada et al., 2024). In January 2022, a self-administered questionnaire with a unique identification number was mailed to 3742 individuals aged 65–84 years, living independently, and registered with an address in two areas targeted by the project as of October 2021.

**Figure 1:**
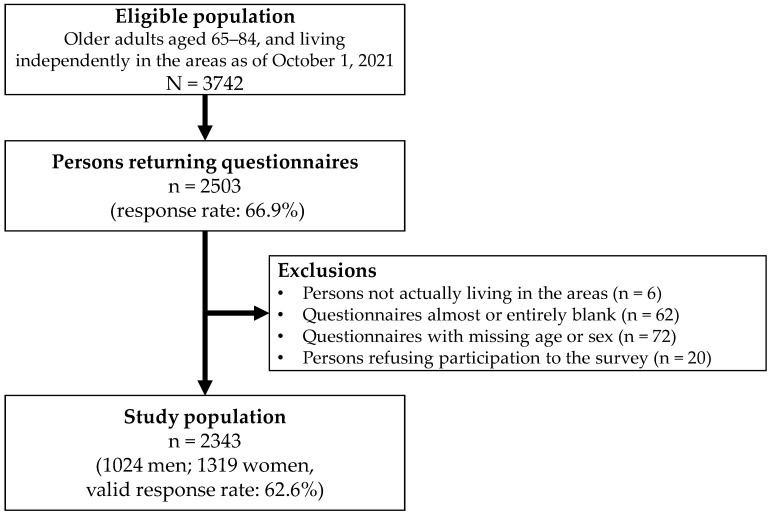
Study Flowchart. Note: The exclusion criteria were applied sequentially from top to bottom; therefore, the numbers in each exclusion category do not overlap.

Of the 3742 individuals invited to participate, 2503 returned the questionnaires (response rate: 66.9%). After excluding participants who did not live in the target area (*n* = 6), returned either almost or entirely blank questionnaires (*n* = 62), had missing identification labels (*n* = 72), or refused to participate (*n* = 20), a final sample of 2343 older adults was included in the analysis (valid response rate: 62.6%; Figure 1).

Informed consent was obtained using a questionnaire administered by the municipal government. While the municipality maintained a correspondence table linking personal identifiers to the study data, researchers received only anonymized data after the participants were given the opportunity to opt out. This procedure was approved by the University of Electrocommunications Ethics Committee (approval number: 21068).

### 2.2. Measures

#### 2.2.1. Subjective Well-Being

Subjective well-being was assessed using the Japanese version of the World Health Organization (WHO) Five Well-Being Index (Awata et al., 2007a, 2007b). This scale consists of five items, with total scores ranging from 0 to 25. Higher scores indicate better mental well-being. The internal consistency of the scale in the present sample was high, with a Cronbach’s alpha of 0.89 (Awata et al., 2007b).

#### 2.2.2. Internet Use

Internet use was assessed using a 4-point scale measuring frequency: almost daily, 2–3 times per week, several times monthly, or no use (Lam et al., 2020). In this study, internet use refers to routine activities, such as browsing websites and sending or receiving emails (C. Li et al., 2023; P. Li et al., 2023).

To assess the purpose of internet use, the participants were asked to select all applicable activities from a predefined list. This list includes items related to practical (online searching, maps, online shopping, music, or video streaming) and social communication purposes (sending and receiving emails and social networking services) (Jeon & Choi, 2024). Based on their responses, the participants were categorized for analysis as follows:

To explore the combined effects of the frequency and purpose of internet use, we created a single categorical variable representing distinct user patterns. This was based on two dimensions: frequency of use and purpose diversity. First, use frequency was dichotomized into “Sporadic” (monthly or weekly use) and “Daily” (almost everyday use). Second, purpose diversity was dichotomized into “Single-purpose” (practical use only or social communication only) and “Dual-purpose” (use for both practical and social purposes). Additionally, participants who reported “Do not use” for frequency were also categorized as “Do not use” for purpose.

#### 2.2.3. Covariates

Sociodemographic data included age, sex, financial status, employment status, smoking and drinking behaviors, self-reported health status, cohabitation, and social participation. Age was categorized into two groups: 65–74 years and 75–84 years. Financial status was evaluated using a self-rated 5-point Likert scale: “Low,” “Middle-low,” “Middle,” “Middle-high,” and “High” (Sakurai et al., 2019). These were categorized as “distressed,” “average,” or “affluent.” Employment status was dichotomized as “currently employed” or “not employed.” Smoking and drinking status were each dichotomized as “current” or “former/never” users. Self-reported health status was assessed using a 4-point Likert scale ranging from “Poor” to “Excellent” and subsequently dichotomized into “excellent/good” or “fair/poor.” Living arrangements were defined as whether the participant lived alone, and social participation was defined as participation in the community or volunteer groups.

### 2.3. Statistical Analysis

Descriptive statistics were used to summarize participant characteristics. Owing to missing data, we performed multiple imputations using chained equations (MICE) to create 20 imputed datasets, assuming that the data were missing at random (MAR). The imputation model included all variables from the analytical model, including the WHO-5 score, internet use variables, and covariates. All analyses were performed on each imputed dataset separately, and the results were combined using Rubin’s rules (Rubin, 1987) to obtain valid statistical inferences that properly reflected the uncertainty due to missing data.

Two separate hierarchical multiple regression analyses were conducted on the pooled data to examine the associations between the frequency and purpose of internet use and WHO-5 scores. In each analysis, the primary independent variable was added in Model 1, age and sex were entered in Model 2, and all covariates were entered in Model 3. The “do not use” group served as the reference category for both the frequency and purpose analyses. Additionally, the adjusted mean WHO-5 scores and their 95% confidence intervals (CIs) were calculated for the five categories of internet use with a combination of frequency and purpose, using a general linear model controlling for all covariates. This two-step analytical strategy was chosen because a single, simultaneous regression model including dummy variables for both frequency and purpose was initially considered but revealed severe multicollinearity.

To address potential selection bias regarding internet use frequency, we conducted a sensitivity analysis using inverse probability of treatment weighting (IPW) based on propensity scores. This analysis was restricted to “Daily use” and “Do not use” groups. Propensity scores were estimated using a logistic regression model, with daily internet use as the outcome and all covariates listed in the full model as predictors. The resulting inverse probability weights were applied to the hierarchical multiple regression model to estimate the association between daily internet use and subjective well-being while accounting for the observed baseline differences between the groups. Furthermore, to explore potential heterogeneity in these associations, we conducted additional exploratory analyses using the original dataset. We examined the interaction effects between internet use (both frequency and purpose) and key demographic variables, including age group, sex, and financial status.

All statistical analyses were performed using IBM SPSS Statistics for Windows, version 29.0 (IBM Corp., Armonk, NY, USA). To reduce the risk of Type I error due to multiple comparisons, we applied the Bonferroni adjustment and adopted a more stringent significance level of *p* < 0.01 for these primary analyses. For all other analyses, *p* < 0.05 was considered statistically significant.

## 3. Results

### 3.1. Participant Characteristics

Table 1 presents the baseline characteristics of the participants. Regarding internet use frequency, 45.2% of the participants reported using the internet daily, whereas 35.2% reported not using it. The most common purpose was practical and social communication (44.9%), followed by a single-purpose use (15.6%). The mean WHO-5 score of the entire sample was 15.0 (SD = 5.4).

### 3.2. Association Between Internet Use and Subjective Well-Being

The results of the hierarchical multiple regression analyses examining the association between internet use and subjective well-being are presented in Table 2.

In the fully adjusted model controlling for all covariates, the frequency of internet use was significantly associated with well-being (*p* for trend <0.001). Daily internet use was associated with significantly higher WHO-5 scores (B = 1.04, 95% CI [0.53, 1.56], *p* < 0.001). In contrast, sporadic use (weekly or monthly) was not significantly associated with a difference in well-being scores (B = −0.05, 95% CI [−0.66, 0.57], *p* = 0.883). The sensitivity analysis using IPW confirmed the robustness of our primary findings. In the IPW-adjusted model, daily internet use remained significantly associated with higher subjective well-being scores compared to non-use (*p* < 0.001).

For internet use purposes, the adjusted model revealed that using the internet for dual purposes (both practical and social) was significantly associated with higher WHO-5 scores than non-use (B = 0.80, 95% CI [0.28, 1.31], *p* = 0.002). Although using the internet for a single purpose (either practical or social only) was not significantly associated with higher well-being (B = 0.55, 95% CI [−0.07, 1.17], *p* = 0.081), a significant positive trend was observed (*p* for trend = 0.003).

We tested for interaction effects between our internet use variables (frequency and purpose) and key demographic characteristics, including age group, sex, and financial status. The analyses revealed no significant interactions between internet use frequency and age group (*p* = 0.423), sex (*p* = 0.922), or financial status (*p* = 0.791). Similarly, no significant interactions were found between internet use purpose and age group (*p* = 0.332), sex (*p* = 0.459), or financial status (*p* = 0.715).

**Figure 2:**
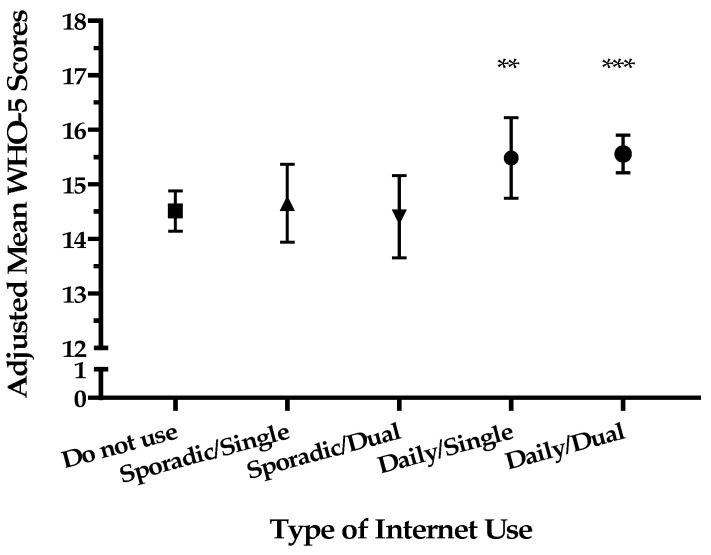
Adjusted mean WHO-5 scores by internet use frequency and purpose. Error bars represent 95% confidence intervals. Means are adjusted for covariates. ** *p* < 0.01, *** *p* < 0.001 compared to the “Do not use” group.

Figure 2 displays the adjusted mean WHO-5 scores for the five distinct patterns of internet use, controlling for all covariates. The “Do not use” group had an adjusted mean score of 14.5 (95% confidence interval [CI] 14.1, 14.8). The scores for the sporadic use groups were similar, with means of 14.6 (95% CI [13.9, 15.3]) for single-purpose use and 14.4 (95% CI [13.6, 15.1]) for dual-purpose use. In contrast, both daily use groups showed markedly higher scores. The “Daily/Single” use group had an adjusted mean score of 15.6 (95% CI [14.9, 16.4]), and the “Daily/Dual” use group had a score of 15.5 (95% CI [15.2, 15.9]). As indicated in Figure 2, both the “Daily/Single” (*p* < 0.01) and “Daily/Dual” (*p* < 0.001) groups had significantly higher mean scores than the “Do not use” group.

## 4. Discussion

This study provides novel evidence of the differential associations between internet use frequency and purpose and subjective well-being among older adults. First, only daily internet use, not sporadic use, was significantly associated with higher well-being compared to non-use. Second, using the internet for dual purposes (both practical and social) was associated with higher well-being, whereas single-purpose use was not significant. Finally, the analysis of combined user patterns revealed that daily use, regardless of purpose diversity, was the primary factor of higher well-being scores than non-use. These results suggest that the benefits of internet engagement are contingent primarily on its regularity, regardless of the diversity of internet use among older adults.

Our findings are consistent with, and extend, the results of a recent comprehensive meta-analysis (Liu et al., 2025) that demonstrated that internet use and its purpose are critical factors, with communication-focused activities yielding the most significant benefits for well-being. However, in their meta-analysis, the authors highlighted a key limitation in the existing literature, noting that their analysis focused on the purposes of internet use, neglecting other dimensions such as the frequency and variety of internet activities. Our study aimed to address this gap. By systematically examining both the frequency (regularity) and purpose diversity (variety) of internet use, we provide a granular understanding of digital behaviors that builds upon the foundational finding that “regular internet use matters.”

Ragnedda (2018) argued that there was a crucial distinction between those who merely access the internet and those who can use it effectively to transform the online experience into something concrete and tangible in their offline lives (Ragnedda, 2018). Our finding that only daily use was beneficial suggests that this regularity is a prerequisite for achieving effective and productive engagement. By contrast, sporadic use may be insufficient to build and maintain the capital necessary to yield tangible well-being outcomes, a finding that resonates with longitudinal evidence showing that low-frequency use is associated with a decline in life satisfaction (Lam et al., 2020).

Our findings also contribute to resolving the “Internet Paradox”—the conflicting reports of both positive and negative effects of internet use on well-being. While some studies have linked high internet use to lower quality of life and health (Y. Li et al., 2025; Vidiasratri & Bath, 2022), our results suggest this is contingent on the patterns of use. Longitudinal evidence has shown that while communication-focused activities are beneficial, information seeking can be detrimental to life satisfaction (Lam et al., 2020). Our key finding that dual-purpose use is the most beneficial suggests a potential buffering effect, in which the positive outcomes of one type of engagement may counteract or mitigate the potential negative effects of another. This synergistic effect may be particularly relevant in the Japanese context. This aligns with Japanese studies suggesting that ICT use indirectly enhances well-being by augmenting social capital (Kokubun et al., 2022). Furthermore, this multifaceted engagement with internet use may contribute to maintaining physical and cognitive functioning (Hirose et al., 2024; Nakagomi et al., 2022; Tajika et al., 2024).

These findings have significant practical and policy-making implications. Digital inclusion initiatives for older adults must evolve from providing access and basic skill training to fostering digital capital formation. Given that regularity of use is the primary factor of well-being, the foremost goal should be to help older adults integrate internet use into their daily routines. This requires creating programs that showcase the immediate benefits of diverse and purposeful use, thereby fostering an intrinsic motivation for sustained engagement. Note that the observed difference in the WHO-5 score was approximately 1.0 point on the 0–25 scale, and this effect size may appear modest. A systematic review on the WHO-5 index highlighted that a 2.5-point difference is considered a clinically relevant threshold for improvement in intervention studies for depressive symptoms (Domenech et al., 2025). While the effect observed in our study does not reach this threshold for a clinically dramatic gain for an individual, it needs to be framed as a meaningful population-level shift. Furthermore, the results to explore potential heterogeneity suggest that the observed main effects of internet use are consistent across these demographic subgroups in our sample. However, this finding should be interpreted with caution. Previous research suggests that the utility of internet use for well-being is not uniform across all older adults. Specifically, the positive effects appear to be non-significant for those in socioeconomically disadvantaged positions (Zhang et al., 2025). Therefore, digital inclusion initiatives must go beyond merely promoting daily use; they should be designed with tailored support to ensure that technology serves to narrow, rather than widen, existing health inequalities.

This study has several limitations. First, as this was an exploratory study, post hoc hypotheses regarding the primacy of regularity were generated. Consequently, our findings should be interpreted as hypothesis-generating rather than confirmatory; they require future validation. Second, the cross-sectional design precludes causal inference due to the possibility of reverse causality; it is equally plausible that individuals with higher well-being are more likely to use the Internet daily. While statistical methods like instrumental variable analysis can address endogeneity, identifying a valid instrumental variable was not feasible in our dataset. Third, the generalizability of our findings may be limited. The sample was drawn from only two areas of a single city, and the valid response rate of 62.6% raises the possibility of selection bias, as participants may be healthier or more socially active than non-respondents. Fourth, data were collected in January 2022 during the COVID-19 pandemic, a unique context that may have influenced both internet use patterns and well-being in atypical ways. Fifth, our study has measurement limitations. Our measures relied on self-report, which is prone to bias, and our assessment of internet use was rudimentary, lacking details on the total duration or specific activities and platforms. Furthermore, because all data were collected from the same source at a single point in time, common method bias (CMB) could be a concern. The Harman’s single-factor test suggested that no single factor accounted for the majority of the variance (30.5%), but this cannot entirely rule out the influence of CMB. Notably, our analysis did not differentiate between practical and social purposes of internet use, which might obscure their individual associations with well-being. Finally, we did not control for potentially critical confounders, such as educational attainment, cognitive function, disorders, quality of social interactions, and emotional aspects. Future studies should address these limitations by employing longitudinal designs, more representative samples, and granular measures to establish causality. Such studies should aim to include objective data on internet use (e.g., log data) and control for key variables like educational background and cognitive function.

## 5. Conclusions

This study found that, for older adults, regularity of internet use is more strongly associated with subjective well-being than diversity of purpose. While versatile and multipurpose engagement is beneficial, a key factor for realizing the internet’s positive potential appears to be its integration into daily life. This finding provides crucial insight into the mechanics of digital capital formation, suggesting that the accumulation of capital through sustained practice is the foundational step upon which capital quality can be built. These results represent a call for digital inclusion policies to shift the focus from merely providing access to fostering the skills and motivation necessary for regular, meaningful engagement. As societies continue to age and digitize, ensuring that older adults can fully and consistently participate in digital life is not just a matter of reducing inequality but a critical strategy for promoting successful aging for all.

## Figures and Tables

**Table 1:** Participants’ characteristics.

Variables	n	%	Missing, n	%
Age			0	0.0
65–74	1232	52.6		
75–84	1111	47.4		
Sex			0	0.0
Male	1024	43.7		
Female	1319	56.3		
Alcohol (Current)	1307	55.8	10	0.4
Smoking (Current)	215	9.2	12	0.5
Employment status (Yes)	776	33.1	30	1.3
Financial status			17	0.7
Distressed	379	16.2		
Average	888	37.9		
Affluent	1059	45.2		
Self-rated health (good to excellent)	1914	81.7	28	1.2
Living alone	454	19.4	17	0.7
Social participation	1032	44.0	30	1.3
Internet use frequency			75	3.2
Do not use	824	35.2		
Sporadic	385	16.4		
Daily	1059	45.2		
Internet use purpose			103	4.4
None	824	35.2		
Single use	365	15.6		
Dual use	1051	44.9		
	mean	SD	Missing, n	%
WHO-5	15.0	5.4	68	2.9

Note: Values are presented as n (%) or mean (SD). The percentages were calculated based on the total number of cases for each variable. The data were from the original dataset (n = 2343).

**Table 2:** Association between Internet Use Frequency, Purpose, and Well-Being.

	Crude Model		Age, Sex-Adjusted Model		Multivariate-Adjusted Model	
	**B (95% CI)**	***p*-value**	**B (95% CI)**	***p*-value**	**B (95% CI)**	***p*-value**
Internet Use Frequency						
Do not use	Reference		Reference		Reference	
Sporadic use	0.42 (−0.23–1.08)	0.204	0.54 (−0.12–1.20)	0.106	−0.05 (−0.66–0.57)	0.883
Daily use	1.99 (1.49–2.49)	<0.001	2.26 (1.73–2.79)	<0.001	1.04 (0.53–1.56)	<0.001
Internet Use Purpose						
Do not use	Reference		Reference		Reference	
Single use	0.95 (0.3–1.61)	0.005	1.14 (0.47–1.8)	<0.001	0.55 (−0.07–1.17)	0.081
Dual use	1.82 (1.31–2.32)	<0.001	2.04 (1.51–2.57)	<0.001	0.80 (0.28–1.31)	0.002

Note. B = unstandardized coefficient; CI = confidence interval. All models included only Internet use frequency or purpose. The adjusted model was controlled for age, sex, alcohol consumption, smoking status, employment status, economic status, self-rated health, living alone, and social participation. The results are based on pooled data from 20 imputations. In the multivariate-adjusted model, the effect size (partial eta squared, ηp^2^) for the overall factor of Internet Use Frequency was 0.010, and for Internet Use Purpose it was 0.005. These effect sizes were calculated based on the original dataset.

## Data Availability

No new data were created or analyzed in this study. Data sharing is not applicable to this article.

## Abbreviations

The following abbreviations are used in this manuscript:

WHO-5	WHO-Five Well-Being Index
WHO	World Health Organization
CDC	Chofu-Digital-Choju
CI	Confidence Interval
SD	Standard Deviation
MICE	Multiple Imputation by Chained Equations
